# When Education Pays Less: Psychological Well-Being, Financial Strain, and Social Support Among Deaf Adults

**DOI:** 10.3390/ejihpe16070103

**Published:** 2026-07-17

**Authors:** Jeffrey Levi Palmer, Carrie Lou Bloom, Mary Sanderson, Linling Shen

**Affiliations:** Department of Special Education, The University of Texas at Austin, Austin, TX 78712, USA; carrielou@utexas.edu (C.L.B.); mary.sanderson@austin.utexas.edu (M.S.); linling.shen@utexas.edu (L.S.)

**Keywords:** deaf, educational attainment, social determinants of health, well-being

## Abstract

Education is a well-established determinant of health and well-being, yet its benefits may not be equally distributed across populations. This study examines whether educational attainment predicts psychological well-being, financial difficulty, and social support among deaf adults and whether these associations differ from those observed among hearing adults. Using nationally representative data from the U.S. Household Pulse Survey, we analyzed a weighted sample of adults aged 25–54 (*N* = 36,810), employing multivariate linear regression models that included education, hearing status, their interaction, and demographic covariates. Higher education was generally associated with more favorable outcomes among deaf adults, particularly in psychological well-being and social and emotional support, though effects were modest and less consistent for financial difficulty. Hearing adults reported significantly better outcomes across all domains, and interaction effects indicated that the benefits of education were significantly larger for hearing adults than for deaf adults. These findings suggest that while education remains beneficial for deaf people, its protective effects are limited, likely due to persistent structural and communication barriers. Given the importance of education, improving access alone will not eliminate disparities in well-being without concurrent efforts to address structural and social inequities.

## 1. Introduction

Education is one of the strongest and most consistent social determinants of health and well-being, with higher educational attainment linked to better psychological, economic, and social outcomes across the life course. Yet, deaf adults continue to experience substantial disparities in employment, financial stability, and mental health compared with hearing adults, even when they achieve similar levels of education. Research has shown that educational attainment improves outcomes for deaf adults, but the magnitude of these benefits appears smaller than those observed in the general population. Prior studies have typically focused on single outcomes, treated education as a control variable, or relied on small or non-representative samples, leaving unanswered questions about whether education functions as a similarly strong predictor of overall well-being for deaf and hearing adults.

Understanding whether the benefits of education extend equally to deaf adults is essential for evaluating education as a health-promoting resource and for identifying structural barriers that may constrain its positive impact. The present study uses nationally representative data to examine the association between educational attainment and well-being outcomes among deaf adults and assesses whether these associations differ from those observed among hearing adults.

## 2. Literature Review

Research consistently shows that education plays a central role in shaping health, economic stability, and overall well-being. A recent global meta-analysis of 59 studies found a robust and consistently positive association between educational attainment and multiple dimensions of well-being, including higher life satisfaction and emotional well-being (Dong et al., 2025). Extensive epidemiologic evidence further demonstrates a graded relationship between educational attainment and life expectancy, morbidity, health behaviors, employment, and economic security. Reflecting this evidence base, The Lancet Public Health (2020) characterizes education as “the most important modifiable social determinant of health,” underscoring both its broad influence and its relative neglect in public health policy and research.

Despite the strength of this association in the general population, the benefits of education are not experienced equally across social groups. Prior research shows that educational attainment does not always confer the same health and well-being benefits across socioeconomic status, race and ethnicity, gender, and disability, suggesting that structural barriers can constrain the protective effects of education even at comparable levels of attainment. These patterns challenge the assumption that education functions as a universally health-promoting resource and highlight the need to examine how its benefits vary across marginalized populations.

Deaf adults represent a population for whom the relationship between education and well-being remains underexplored. Research consistently shows that deaf adults report lower psychological well-being, greater financial strain, and weaker social support than their hearing peers (McAbee et al., 2017; Crowe, 2019; Aldalur & Pick, 2023). These disparities intersect with persistent inequalities in educational attainment: only 23.9% of deaf adults hold a bachelor’s degree or higher, compared to 39.1% of hearing adults (Bloom et al., 2026). For the relatively small share of deaf adults who do attain higher education, existing studies document continued disparities in employment and health outcomes when comparing deaf and hearing adults with similar levels of education (e.g., McKee et al., 2014; Engelman et al., 2021; Bloom et al., 2026).

To understand why education so powerfully shapes health and well-being and why its benefits may vary across populations, P. Braveman (2023) offers a clear conceptual model illustrating three major and interrelated pathways through which education influences health:

(1) Health knowledge, literacy, and health-related behaviors: Education builds cognitive skills, comprehension, and problem-solving abilities that support healthier behaviors such as improved diet, physical activity, and chronic disease management.
(2) Employment, working conditions, and income: Higher education increases access to stable employment, safer working environments, health insurance, and higher earnings, which in turn shape exposure to health-promoting or health-damaging conditions.
(3) Psychosocial resources: Education is associated with a greater sense of control, social standing, and social support, each of which has been linked to improved stress responses and better health outcomes.

These pathways highlight why education is such a powerful predictor of health and well-being across the life course. At the same time, the protective effects of education are not uniform across populations. Studies consistently document diminished health and economic returns to education for racial minority groups, driven by structural factors such as labor-market discrimination, unequal educational quality, and residential segregation (P. A. Braveman et al., 2005; Chiswick, 2020). Variability in the association between education and well-being has also been observed across gender and socioeconomic groups, with psychosocial and mortality-related benefits differing by subgroup (Turiano et al., 2014; Assari, 2017a, 2017b).

These findings highlight the importance of exploring the relationship between education and well-being for populations who have faced longstanding structural barriers, such as deaf adults. For deaf people, the health-related benefits typically associated with educational attainment may be limited or disrupted by barriers in communication access, labor market discrimination, limited social capital, or the quality and accessibility of educational experiences. Understanding how education functions within this population is essential for accurately assessing the role of education in shaping health and for revealing opportunities to disrupt the processes that reproduce inequities.

### 2.1. Well-Being Among Deaf Adults

Deaf adults report lower well-being across multiple psychological, social, and economic domains compared with hearing adults. Across national surveys and targeted studies, deaf adults consistently report lower psychological well-being, higher levels of financial strain, and weaker perceived social support (McAbee et al., 2017; Crowe, 2019; Aldalur & Pick, 2023). These disparities appear robust across different samples and measurement strategies, suggesting that lower well-being among deaf adults is not limited to any single outcome or context.

Research further indicates that well-being among deaf adults is shaped by factors that extend beyond traditional socioeconomic indicators. Qualitative and mixed-methods studies highlight the importance of communication access, social belonging, and culturally aligned environments as central contributors to psychological and social well-being (McAbee et al., 2017; Crowe, 2019). In quantitative analyses, communication experiences often emerge as stronger correlates of well-being than income or educational attainment alone. For example, in a large survey of Deaf ASL-using adults, communication access at work and school was more strongly associated with happiness than income or education, and high levels of well-being were widely reported among those with strong connections to the Deaf community (Crowe, 2019).

Structural and interpersonal barriers further contribute to disparities in well-being. Acculturative stress arising from marginalization within both hearing and deaf contexts has been linked to lower psychological well-being and increased symptoms of depression and anxiety (Aldalur & Pick, 2023). These findings underscore the role of broader social and structural conditions in shaping well-being outcomes among deaf adults.

### 2.2. Educational Attainment Among Deaf Adults

Education generally improves outcomes for deaf adults. Postsecondary education is associated with higher employment rates, higher wages, and greater civic and community engagement among deaf young adults (Bloom et al., 2026; Palmer et al., 2020). Educational attainment is also linked to reduced material hardship: deaf adults with lower education levels are more likely to experience food insecurity, while college-educated deaf adults are disproportionately represented among the food-secure (Engelman et al., 2021). In the domain of physical health, higher education, but not higher income, has been found to be protective against cardiovascular risk among Deaf ASL users, suggesting that education may provide access to health knowledge and preventive behaviors that income alone cannot offer (McKee et al., 2014). Mental health patterns provide further nuance: in national data, deaf adults report higher rates of depression and anxiety diagnoses than hearing adults, and among deaf adults, those with more education are more likely to receive a diagnosis, likely because they have better communication access, greater ability to advocate for care, and more consistent interaction with healthcare providers (Kushalnagar et al., 2019). Despite these benefits, studies suggest that returns to education are smaller for deaf adults, likely due to workplace communication barriers, reduced promotion opportunities, and systemic inequities documented across national datasets and postsecondary outcome reports (Palmer et al., 2020; National Deaf Center on Postsecondary Outcomes, 2026). One study found no significant association between education and psychological distress among deaf adults when education was treated only as a control variable, reinforcing the need for models that directly test whether education’s effects differ between deaf and hearing people (Aldalur & Pick, 2023; Assari et al., 2024).

### 2.3. Study Purpose

Across populations, education is a strong determinant of health and well-being, yet its benefits are often smaller for groups facing structural barriers. Although several studies document associations between education and employment, material hardship, or health for deaf adults, most have not directly tested whether educational attainment predicts well-being to the same extent as it does for hearing adults. Education is also frequently included only as a control variable, leaving the strength and shape of its association with well-being understudied. To address this gap, the present study examines whether the relationship between educational attainment and well-being differs for deaf and hearing adults. The guiding research question is as follows: To what extent does educational attainment predict well-being among deaf adults, and does this association differ from that observed among hearing adults?

## 3. Materials and Methods

### 3.1. Data Source

During spring 2020, the U.S. Census Bureau and five federal agencies launched the Household Pulse Survey (HPS) to measure the pandemic’s impact on U.S. households. The HPS uses a large, nationally representative sample to gather data. Households are invited to participate through email and text message outreach, with initial messages sent during the workweek and reminder notices delivered to non-respondents. The survey itself was administered online. For this study, we used data from Phase 4 of the HPS, which includes nine cycles collected between January and September 2024. This phase was selected because it contained the elements needed for our analysis and was the most recent available at the time. By 2024, the U.S. had transitioned out of the COVID-19 emergency period and returned to more typical social and economic conditions, so the data from this phase are more likely to reflect routine patterns rather than pandemic-related disruptions.

### 3.2. Analytic Sample

The analytic sample was restricted to adults aged 25–54 to ensure that most respondents had completed their formal education while remaining in the core working-age population and to reduce the likelihood that hearing loss is age-related. Survey weights were applied in all analyses to account for the complex sampling design and to produce population-representative estimates.

### 3.3. Measures

#### 3.3.1. Deaf-Hearing Groups

Hearing status was measured using the HPS functional hearing-difficulty item, which asks, “Do you have difficulty hearing, even when using a hearing aid?” Response options included no difficulty, some difficulty, a lot of difficulty, and cannot do at all. Following prior disability-focused work using this dataset (e.g., Morales, 2025; Surfus, 2025), respondents selecting “a lot of difficulty” or “cannot do at all” were classified as deaf for analytic purposes. Those reporting “some difficulty” were excluded from the analytic sample. Thus, analyses compared adults reporting no hearing difficulty (hearing) with adults reporting substantial hearing difficulty (deaf). This grouping aligns with prior work (Mitchell & Young, 2023) showing that adults reporting the most severe hearing difficulties form a distinct analytic category, particularly in communication and sign-language use patterns.

It is important to note that this operationalization captures a functionally heterogeneous deaf group. Respondents classified as deaf may include culturally Deaf ASL users, individuals who use hearing aids or cochlear implants and function primarily in spoken language environments, and those with age-related hearing loss. These subgroups likely differ in their access to social networks, communication resources, and cultural identity, factors that may shape psychological well-being and social support in ways the current analysis cannot disentangle. At the same time, examining deaf adults as a group captures their shared experience of navigating predominantly hearing educational, workplace, and social environments. This aggregated perspective offers its own analytic value, particularly for understanding population-level disparities and informing broad policy and accessibility efforts.

#### 3.3.2. Education Level

Education level was based on self-reported education level and was categorized into three groups: high school diploma or less, attended some college but did not receive a degree, or any postsecondary degree, including a bachelor’s degree or higher.

#### 3.3.3. Well-Being Outcomes

Overall well-being was examined across three domains: (1) psychological well-being, (2) financial difficulty, and (3) social and emotional support. Psychological well-being was measured using four HPS items (ANXIOUS, WORRY, INTEREST, and DOWN) that asked respondents how often they experienced anxiety, worry, loss of interest, and depressed mood during the previous two weeks. These items represent distinct but related aspects of emotional functioning, and taken together, they offer a broader and more reliable view of psychological well-being than any single item could provide. Internal consistency indices and confirmatory factor analysis supported the interpretation of these items as a single underlying construct, and they were modeled as a latent psychological well-being factor in the analyses.

Financial difficulty was measured using a single HPS item, EXPNS_DIF, which asked respondents how difficult it was to pay for usual household expenses in the previous seven days. The item captures a clear and unambiguous aspect of household financial strain. Responses were reverse-coded so that higher values reflected lower financial strain.

Social and emotional support was measured using a single HPS item, SOCIAL1, which asks respondents how often they received the social and emotional support they needed during the previous two weeks. Although the survey includes several items related to social interaction, these questions tap into different types of social contact rather than a single, unified construct of perceived support. In addition, some items may not resonate well with deaf respondents, for example, questions about “calling people on the telephone.” When we attempted to combine selected items into a composite variable, analyses of internal consistency and confirmatory factor analysis indicated that the items did not function together as a coherent scale.

Respondents missing information on all well-being outcomes were excluded from the relevant analyses, resulting in an unweighted analytic sample of N = 36,810.

#### 3.3.4. Covariates

Age, gender, and race/ethnicity were included as covariates in all multivariate regression models. Age was measured in years as a continuous variable. Gender was based on the HPS self-reported gender at birth item (male, female). Although the HPS includes a gender identity item as part of the Sexual Orientation and Gender Identity (SOGI) module introduced in Phase 3.2, this variable exhibited substantial missingness in Phase 4 and was therefore not included in the analytic models. Race/ethnicity was measured using the HPS racial/ethnic categories and represented with indicator variables for Hispanic, Black, Asian, and Other, with White serving as the reference category. These covariates were included to adjust for demographic factors known to be associated with socioeconomic resources and well-being outcomes in national survey data.

### 3.4. Statistical Analysis

**Figure 1:**
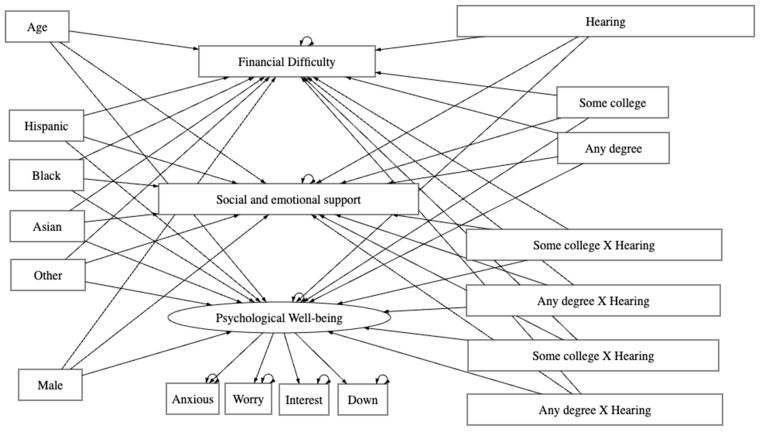
Multiple linear regression model. *Note*. Reference group: White, female, deaf adults with a high school education or less.

To address the research question asking to what extent education level predicts well-being outcomes among deaf adults aged 25–54 in comparison to hearing adults, we estimated multivariate linear regression models using Mplus (Version 8.3). All analyses incorporated survey weights to account for the complex sampling design and to produce population-representative estimates. Rather than fitting separate regression models for each outcome, the three well-being outcomes were modeled jointly within a multivariate regression framework. This approach allows residual correlations among outcomes to be freely estimated, accounting for shared unobserved factors and improving statistical efficiency. The overall model specification is illustrated in Figure 1.

Each outcome was regressed on education level, hearing status, their interaction, and a set of covariates. The model for each outcome variable can be expressed as

*Y_k_* = *β*_0*k*_ + *β*_1*k*_ (*Education*) + *β*_2*k*_ (*Heading Status*) + *β*_3*k*_ (*Education* × *Hearing Status*) + *β*_4–6*k*_ (*Covariates*) + *ε_k_* (1)

where *Y*_*k*_ denotes outcome *k*, and covariates include age, gender, and race/ethnicity, while *ε*_*k*_ represents the outcome-specific residual. The reference group for interpretation consisted of deaf adults who had a high school education or less. Accordingly, coefficients for education *β*_1*k*_, hearing status *β*_2*k*_, and their interaction *β*_3*k*_ represent deviations from this reference group. Interaction terms were used to evaluate whether associations between education and well-being outcomes differed between deaf and hearing adults.

## 4. Results

Table 1 presents weighted demographic characteristics of the analytic sample by hearing status. The weighted sample included 248,445 hearing adults and 2954 deaf adults. No missing data were observed for demographic variables. Deaf adults were more likely to be male (54.6%) than hearing adults (47.5%). Hearing and deaf adults were predominantly White (55.3% and 50.7%, respectively). Educational attainment differed by hearing status. Nearly half of hearing adults (49.3%) had attained a postsecondary degree, compared to 31.6% of deaf adults, while most deaf adults (48.7%) reported that they had attained some college but did not receive a degree.

Table 2 reports results from the multivariate linear regression models examining associations between education level, hearing status, and three well-being outcomes while adjusting for age, gender, and race/ethnicity. Due to the complexity of the model, Mplus was unable to compute traditional global fit indices (e.g., CFI, TLI), which can occur when the baseline model fails to converge. However, the SRMR indicated acceptable model fit (SRMR = 0.023), consistent with recommended thresholds (SRMR < 0.08; Hu & Bentler, 1999), supporting the adequacy of the multivariate regression specification.

### 4.1. Main Effects of Education

For deaf adults, when compared to having a high school education or less, having any postsecondary degree was associated with significantly higher psychological well-being (β = 0.10, SE = 0.04, *p* = 0.01), whereas attending some college without earning a degree was not significantly associated with higher psychological well-being (β = 0.05, SE = 0.05, *p* = 0.34). Additionally, no significant difference was observed between individuals with some college and those with a postsecondary degree.

For financial difficulty, deaf adults with any postsecondary degree reported significantly lower levels of difficulty than those with some college (β = 0.13, SE = 0.04, *p* = 0.001), although differences relative to high school or less were not statistically significant. In contrast, education was strongly associated with social and emotional support: both some college (β = 0.21, SE = 0.05, *p* < 0.001) and any degree (β = 0.19, SE = 0.05, *p* < 0.001) were associated with significantly higher support compared to high school or less. No significant difference was observed between participants with some college education and those who had any postsecondary degree.

### 4.2. Main Effects of Hearing Status

Hearing adults reported significantly better outcomes than deaf adults across all three domains. Specifically, hearing adults exhibited higher psychological well-being (β = 0.82, SE = 0.03, *p* < 0.001), lower financial difficulty (β = 0.39, SE = 0.03, *p* < 0.001), and greater social and emotional support (β = 0.48, SE = 0.04, *p* < 0.001), conditional on education and covariates.

### 4.3. Education × Hearing Interactions

**Figure 2:**
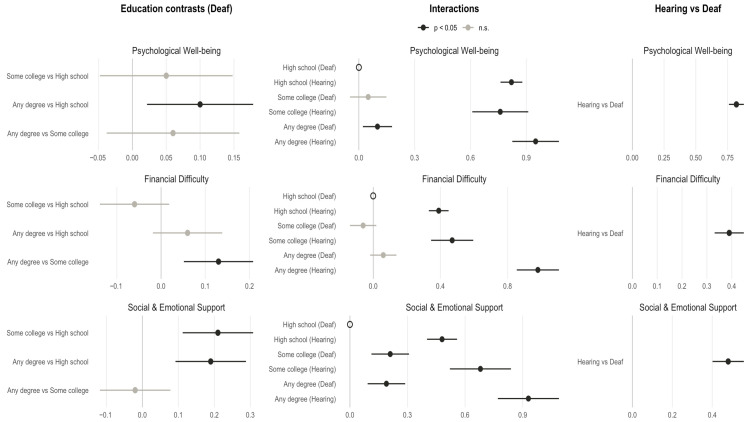
Predicted differences by education level and hearing status.

Interaction effects indicated that the association between education and well-being differed by hearing status (see Figure 2). For psychological well-being, the interaction between hearing status and some college was negative and statistically significant (β = −0.11, SE = 0.05, *p* = 0.03), indicating that the psychological well-being advantage associated with some college was smaller for hearing adults than for deaf adults. Hearing adults report higher psychological well-being at every education level (Figure 2); the negative coefficient reflects a narrowing of this gap at the some-college level relative to high school or less, suggesting that some college may be comparatively more beneficial for deaf adults’ psychological well-being than for hearing adults at that education level. No significant interaction was observed for having any degree.

For financial difficulty, both interaction terms were positive and statistically significant. The association between education and financial outcome was stronger for hearing adults than for deaf adults, both for some college (β = 0.14, SE = 0.04, *p* < 0.001) and for any degree (β = 0.53, SE = 0.04, *p* < 0.001). These findings suggest that the financial advantages associated with having some college experience or attaining a postsecondary degree are smaller among deaf adults than among hearing adults.

Similarly, for social and emotional support, the interaction between hearing status and any degree was positive and significant (β = 0.26, SE = 0.05, *p* < 0.001), whereas the interaction with some college was not significant. Together, these results indicate that higher education is associated with smaller gains in social and emotional support among deaf adults than among hearing adults.

## 5. Discussion

The present study examined whether education level predicts psychological well-being, financial difficulty, and social and emotional support among deaf adults and whether these associations differ from those observed among hearing adults. Several clear patterns emerged. First, higher educational attainment was generally associated with more favorable well-being outcomes among deaf adults, particularly in psychological well-being and social and emotional support. Second, hearing adults consistently reported better outcomes across all three domains, even after accounting for education and demographic factors. Third, significant interaction effects indicated that the benefits of education were not equally distributed: hearing adults experienced larger gains from higher education, especially in the financial domain. All in all, these findings suggest that although education improves outcomes for deaf people, it may not be sufficient to overcome the persistent structural and social barriers that shape their experiences.

### 5.1. Relevance to Previous Literature

Across a wide body of research, education is widely regarded as a key pathway to improved economic stability, health, and overall well-being. However, these benefits are not distributed equally. Studies consistently show that individuals from structurally marginalized groups—including racialized minorities, immigrants, and people with disabilities—often experience diminished returns on educational attainment compared to their white, non-disabled counterparts (Assari, 2017a, 2017b; Assari et al., 2024; P. A. Braveman et al., 2005; Turiano et al., 2014). That is, while higher education confers advantages, it does not fully offset existing structural inequalities in labor markets, social institutions, and access to opportunity. The present findings situate deaf adults within this broader pattern. Although education remains beneficial, it does not appear sufficient to level the playing field, as systemic barriers continue to shape outcomes. In this way, our results extend the literature on educational returns to highlight how structural inequities persist even among highly educated deaf individuals.

The financial gap is especially striking given that postsecondary degrees confer the largest financial returns for hearing adults yet appear to close relatively little of the financial difficulty gap for deaf adults. Several mechanisms likely contribute to this pattern. Research on deaf college graduates documents persistent barriers to career advancement, including workplace communication barriers that limit participation in informal networks (Cawthon et al., 2016) and decision-making spaces, reduced access to mentorship when hearing supervisors and colleagues lack familiarity with deaf people (Braun et al., 2017), and promotion ceilings that emerge not at entry-level employment but over the longer arc of a career (Kelly et al., 2016). Kelly et al. (2016) found that while young deaf and hearing graduates occupied similar job roles early in their careers, hearing graduates were disproportionately represented in managerial and high-level positions a decade later, suggesting that advancement barriers operate over time. This pattern of limited career advancement is not new; Welsh and Walter (1988) documented a similar lack of vertical mobility among deaf college graduates decades earlier, suggesting that structural barriers to advancement have persisted despite increases in educational attainment. Underemployment, whereby deaf degree-holders work in roles that do not fully utilize their qualifications, may further suppress the financial returns to education for deaf adults (Palmer et al., 2020). The evidence points to a labor market that limits how educational credentials translate into financial stability for deaf adults, regardless of what they achieve academically.

Among deaf adults, some college experience was associated with meaningfully higher social and emotional support, comparable in magnitude to completing a degree, and no significant interaction with hearing status was observed for this group. This pattern suggests that partial postsecondary exposure may confer social network benefits for deaf adults even without degree completion. Postsecondary environments, even when not completed, provide access to peers, support services, and community connections that may persist beyond enrollment. This interpretation is consistent with Palmer et al. (2020), who found that postsecondary non-completers did not differ significantly from graduates on most social and community engagement outcomes, with both groups differing primarily from those who never attended. The high proportion of deaf adults in the present sample reporting some college but no degree, nearly half of the deaf sample compared to fewer than one in five hearing adults, underscores the relevance of this finding. This pattern aligns with prior research documenting persistent degree-completion gaps for deaf individuals (Bloom et al., 2026; Garberoglio et al., 2021; Palmer et al., 2020), suggesting that non-completion, rather than non-enrollment, may be a particularly salient barrier for this population and one that warrants greater attention in both research and policy.

Our results raise the possibility, supported by prior qualitative and survey research, that communication access, social belonging, and culturally aligned environments contribute meaningfully to deaf adults’ psychological and social well-being (Crowe, 2019; McAbee et al., 2017). While the literature suggests that education should enhance psychosocial resources, our findings show only modest gains for deaf adults, indicating that the mechanisms through which education typically improves well-being may not operate as effectively when communication barriers persist. This interpretation aligns with social-determinant models (e.g., P. Braveman, 2023) by demonstrating that the protective pathways of education are contingent on structural accessibility and inclusion. Additionally, heterogeneity within the deaf group may contribute to this pattern. Individuals who are culturally Deaf and community-connected tend to report higher well-being than those who are late-deafened or more socially isolated (Crowe, 2019; McAbee et al., 2017), and aggregating these subgroups may diminish the observed association between education and psychological well-being.

### 5.2. Implications

The pattern of diminished returns to education observed in this study suggests that, for deaf adults, the pathways through which education is expected to improve well-being may be partially disrupted by structural and communication barriers. Although higher education was linked to better psychological well-being and greater social and emotional support, these benefits were modest, and the financial returns to education were especially weak relative to hearing adults. This aligns with broader evidence that marginalized groups often experience reduced economic and psychosocial gains from educational attainment due to inequitable labor market access, discrimination, and limited opportunities for upward mobility. For deaf adults specifically, inaccessible workplaces, attitudes and biases, communication barriers, and reduced social capital likely constrain the typical translation of educational credentials into financial stability and supportive social networks. These findings suggest that education alone is insufficient to activate the full set of health-promoting pathways outlined in social-determinants frameworks (e.g., P. Braveman, 2023), highlighting the need to address the structural conditions that limit how educational attainment can contribute to well-being for deaf adults.

Viewed through P. Braveman’s (2023) pathways framework, the present findings suggest that at least two of the three major pathways through which education typically improves health and well-being appear limited for deaf adults. The employment and income pathway, through which education is expected to translate into stable, well-compensated work, shows the greatest attenuation, as evidenced by the notably weak financial returns to postsecondary education among deaf adults. The psychosocial resources pathway, through which education builds social standing and social support, appears partially diminished, with modest but meaningful gains in psychological well-being and social support that remain substantially smaller than those observed among hearing adults. The health knowledge and behaviors pathway was not directly examined in this study but represents an important avenue for future research.

### 5.3. Limitations

Several limitations should be considered when interpreting these findings. Hearing status was measured through self-reported functional difficulty, which may introduce misclassification and does not capture more nuanced distinctions within deaf populations. The cross-sectional nature of the Household Pulse Survey also limits the ability to infer causality between education and well-being outcomes. Because the survey was administered online, individuals with limited internet access or lower digital literacy may be underrepresented, potentially excluding some of the most marginalized deaf adults. Additionally, the psychological well-being measure relied on a brief four-item scale that may not fully reflect the complexity of mental health experiences. Despite these limitations, the study provides valuable insights into the relationship between educational attainment and well-being outcomes among deaf adults.

## 6. Conclusions

This study provides new nationally representative evidence showing that higher education improves psychological, financial, and social outcomes for deaf adults, yet these benefits remain smaller than those experienced by hearing adults. These findings suggest that expanding educational attainment alone will not eliminate well-being disparities unless the structural, economic, and communication barriers that constrain deaf adults’ opportunities are directly addressed. Improving workplace accessibility, strengthening anti-discrimination protections, and ensuring communication-accessible environments across educational and social institutions may help translate educational achievement into more equitable well-being outcomes. Future work should examine how educational opportunities, communication access, and labor-market experiences interact to shape long-term well-being among deaf adults.

## Figures and Tables

**Table 1:** Participant characteristics and measures (weighted).

	Hearing	Deaf	
Sample size (unweighted)	248,445	2954	
Sample size (weighted)	1,053,149,747	14,971,612	
Characteristic	%	%	
Gender			
Female	52.5	45.4	
Male	47.5	54.6	
Race			
Asian	6.4	3.7	
Black	12.9	9.5	
Hispanic	20.8	26.2	
Other	4.7	9.8	
White	55.3	50.7	
Education			
Any degree	49.3	31.6	
Some college	19.0	48.7	
High school or less	31.7	19.6	
Measure	M (SD)	M (SD)	Missing (%)
Age	38.98 (0.04)	41.00 (0.33)	0
Financial difficulty	1.68 (0.00)	1.00 (0.04)	7.65
Social and emotional support	2.48 (0.01)	1.84 (0.05)	4.40
Psychological Well-being			
Anxious	2.20 (0.00)	1.50 (0.04)	2.40
Worry	2.35 (0.00)	1.59 (0.04)	2.45
Interest	2.46 (0.00)	1.69 (0.04)	2.48
Down	2.43 (0.00)	1.67 (0.04)	2.48

**Table 2:** Multivariate linear regression model.

	Psychological Well-Being				Financial Difficulty				Social and Emotional Support			
	β (SE)	*t*	95% CI	*p*	β (SE)	*t*	95% CI	*p*	β (SE)	*t*	95% CI	*p*
**Education Main Effect**												
Some college vs. High school	0.05 (0.05)	0.96	[−0.05, 0.14]	0.34	−0.06 (0.04)	−1.55	[−0.14, 0.02]	0.12	0.21 (0.05)	4.16	[0.11, 0.31]	<0.001
Any degree vs. High school	0.10 (0.04)	2.52	[0.02, 0.19]	0.01	0.06 (0.04)	1.77	[−0.01, 0.13]	0.08	0.19 (0.05)	3.62	[0.09, 0.29]	<0.001
Any degree vs. Some college	0.06 (0.05)	1.27	[−0.03, 0.15]	0.20	0.13 (0.04)	3.43	[0.05, 0.2]	0.001	−0.02 (0.05)	−0.48	[−0.12, 0.07]	0.63
**Hearing Status Main Effect**												
Hearing vs. Deaf	0.82 (0.03)	24.94	[0.76, 0.89]	<0.001	0.39 (0.03)	14.91	[0.34, 0.44]	<0.001	0.48 (0.04)	11.30	[0.39, 0.56]	<0.001
**Interaction**												
Hearing × education (Some college)	−0.11 (0.05)	−2.18	[−0.21, −0.01]	0.03	0.14 (0.04)	3.30	[0.06, 0.22]	<0.001	−0.01 (0.05)	−0.09	[−0.1, 0.09]	0.93
Hearing × education (Any degree)	0.03 (0.04)	0.76	[−0.05, 0.12]	0.45	0.53 (0.04)	14.44	[0.46, 0.6]	<0.001	0.26 (0.05)	5.05	[0.16, 0.36]	<0.001
**Covariates**												
Age	0.02 (0.00)	78.92	[0.02, 0.02]	<0.001	0.01 (0.00)	23.29	[0.01, 0.01]	<0.001	0.00 (0.00)	8.89	[0, 0]	<0.001
Race: Hispanic vs. White	0.12 (0.01)	25.88	[0.11, 0.13]	<0.001	−0.19 (0.01)	−32.07	[−0.2, −0.17]	<0.001	−0.32 (0.01)	−32.82	[−0.34, −0.3]	<0.001
Race: Asian vs. White	0.20 (0.01)	36.06	[0.19, 0.2]	<0.001	0.16 (0.01)	22.33	[0.15, 0.18]	<0.001	−0.33 (0.01)	−31.38	[−0.35, −0.31]	<0.001
Race: Black vs. White	0.10 (0.01)	16.04	[0.09, 0.12]	<0.001	−0.23 (0.01)	−39.69	[−0.24, −0.22]	<0.001	−0.20 (0.01)	−27.48	[−0.22, −0.19]	<0.001
Race: Other vs. White	−0.07 (0.01)	−10.41	[−0.08, −0.06]	<0.001	−0.21 (0.01)	−22.00	[−0.22, −0.19]	<0.001	−0.18 (0.01)	−17.62	[−0.2, −0.16]	<0.001
Gender: Male vs. Female	0.16 (0.00)	42.77	[0.15, 0.17]	<0.001	0.21 (0.00)	49.73	[0.2, 0.21]	<0.001	−0.13 (0.01)	−27.01	[−0.14, −0.12]	<0.001

Note. The second terms are the reference groups. Bold text indicates section headings within the table.

## Data Availability

The data presented in this study are available on request from the corresponding author.

## Abbreviation

The following abbreviation is used in this manuscript:

HPS	Household Pulse Survey
